# Supplementary material for: Mechanism of sensitivity modulation in the calcium-sensing receptor via electrostatic tuning
Source: Nat Commun. 2022 Apr 22;13:2194. doi: 10.1038/s41467-022-29897-y (PMC9033857; doi:10.1038/s41467-022-29897-y)
Supplement: Supplementary file 1 — Supplementary Information [file 41467_2022_29897_MOESM1_ESM.pdf]

Supplementary Information

Mechanism of Sensitivity Modulation in the Calcium-Sensing Receptor via Electrostatic Tuning

Michael Robert Schamber[1], and Reza Vafabakhsh[1*]

[1] Department of Molecular Biosciences, Northwestern University, Evanston, Illinois, 60208
* Corresponding author.

To whom correspondence should be addressed: reza.vafabakhsh@northwestern.edu

Supplementary Figures

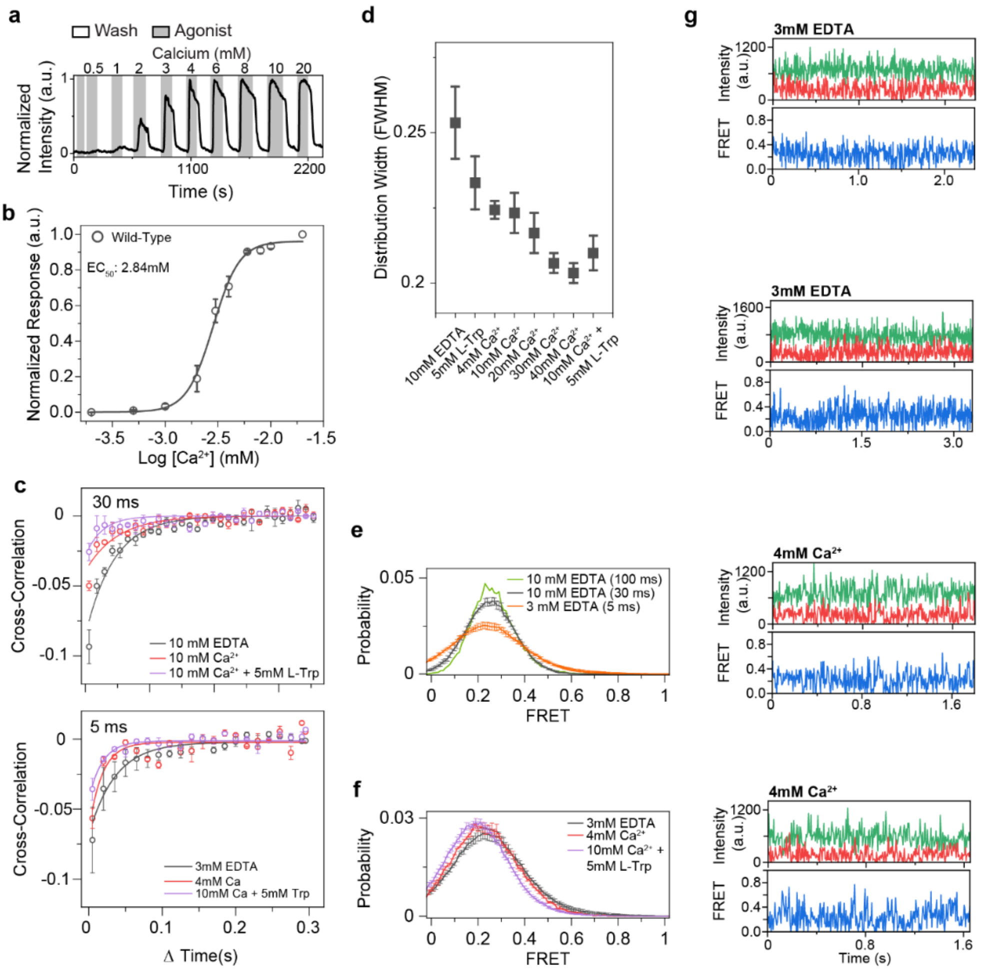

Supplementary Fig. 1 – Functional test of SNAP-CaSR and additional single-molecule characterization at different time resolution (100, 30, 5ms) with sample traces.

a Response profile of HEK293T cells expressing SNAP CaSR. Shaded regions indicate when cells were stimulated with calcium. **b** Dose-response curve for wild-type SNAP-CaSR. Data represents mean ± s.e.m. of n=3 independent biological replicates. Data was fit to the hill equation to calculate EC$_{50}$. **c** Cross-correlation plot quantifying the dynamics of receptors in the presence of 10 mM EDTA, 10 mM Ca^{2+}, or 10 mM Ca^{2+} + 5 mM L-Trp at 5ms (top) and 30ms (bottom) time resolutions. Data represent ± s.e.m of n=3 independent biological replicates. Data was fit to a single exponential decay function. **d** Width of a single gaussian distribution fit to FRET histograms. Data represents the mean ± s.e.m. of n=3 fits to independent biological replicates. **e** smFRET population histogram comparing 100ms, 30ms, and 5ms time resolution in the presence of EDTA. Data represent mean ± s.e.m. of n=3 independent biological replicates for 30 and 5 ms time resolutions and n=1 for 100 ms. **f** smFRET population histogram in the presence of 3 mM EDTA, 10 mM Ca^{2+}, or 10 mM Ca^{2+} and 5 mM L-Trp collected at 5ms time resolution. Data represent mean ± s.e.m. of n=3 independent biological replicates except for 10mM EDTA (100ms), which is Nv=1. **g** Sample single-molecule trace at 5ms time-resolution for SNAP-CaSR in 3 mM EDTA (top) and 4 mM Ca^{2+} (bottom) showing donor (green) and acceptor (red) intensities and corresponding FRET values (blue).

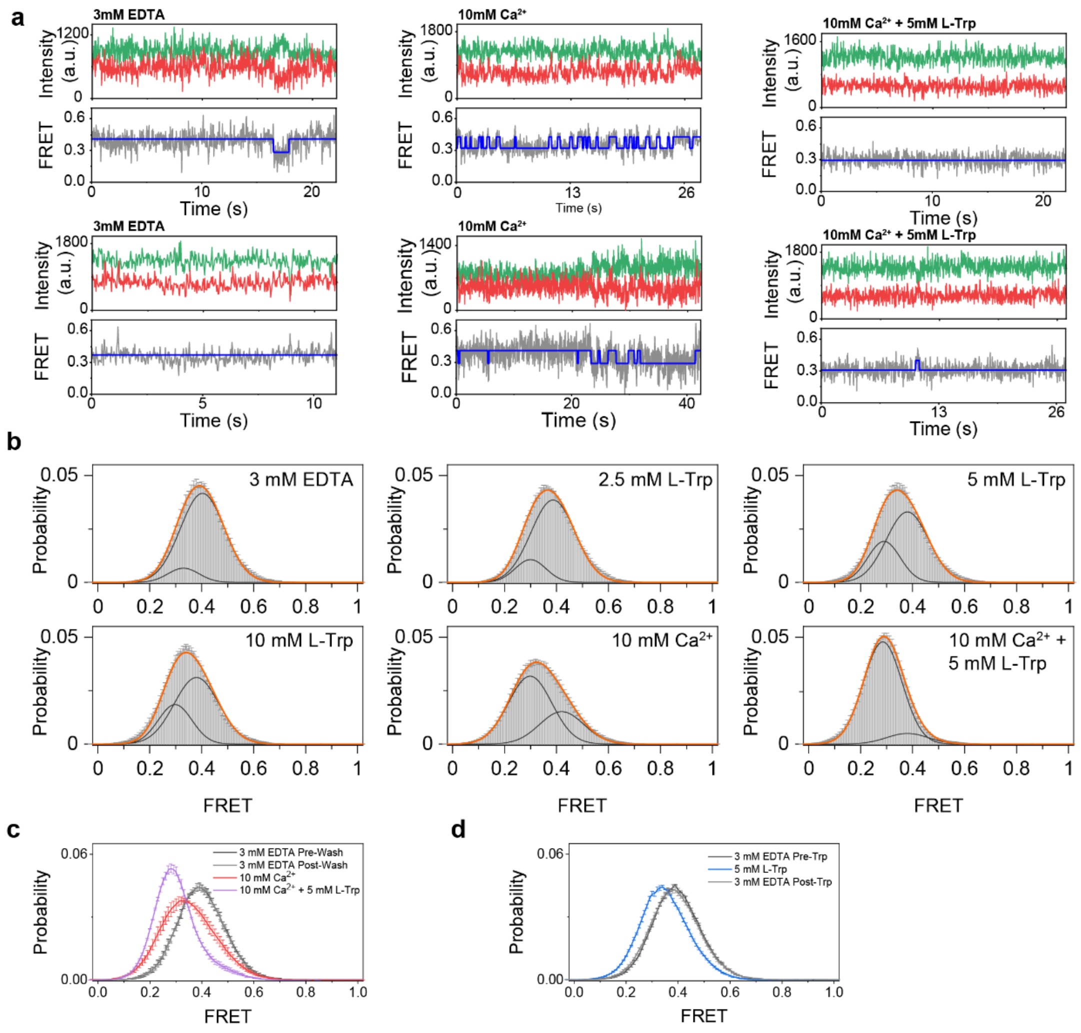

Supplementary Fig. 2 – Sample traces for D451UAA CaSR.

a Sample single molecule traces of D451UAA in 3 mM EDTA (top), 10 mM Ca^{2+} (middle), and 10 mM Ca^{2+} + 5 mM L-Trp (bottom) showing donor (green) and acceptor (red) intensities, corresponding FRET values (gray), and idealized FRET trajectory from HMM fit (blue). **b** smFRET population histogram in the presence of 3 mM EDTA, 10 mM Ca^{2+}, 2.5 mM L-Trp, 5 mM L-Trp, 10 mM L-Trp, or 10 mM Ca^{2+} and 5 mM L-Trp. Data represent mean ± s.e.m. of n=3 independent biological replicates. Histograms were fit with two single gaussian distributions (black) centered at 0.41 and 0.29, and the cumulative fit is overlaid (orange). **c** smFRET population histograms comparing CaSR before a 2-hour wash (dark gray), after the 2-hour wash (light gray), and in the presence of 10 mM Ca^{2+} (red) and 10 mM Ca^{2+} + 5 mM L-Trp (purple). Pre and post wash histograms overlap. Data represent ± s.e.m of n=3 independent biological replicates. **d** smFRET population histograms comparing 3 mM EDTA before the addition of L-Trp (dark gray), in the presence of L-Trp (blue), and after L-Trp has been washed out (light gray). Pre and post L-Trp histograms overlap. Data represent ± s.e.m of n=3 independent biological replicates.

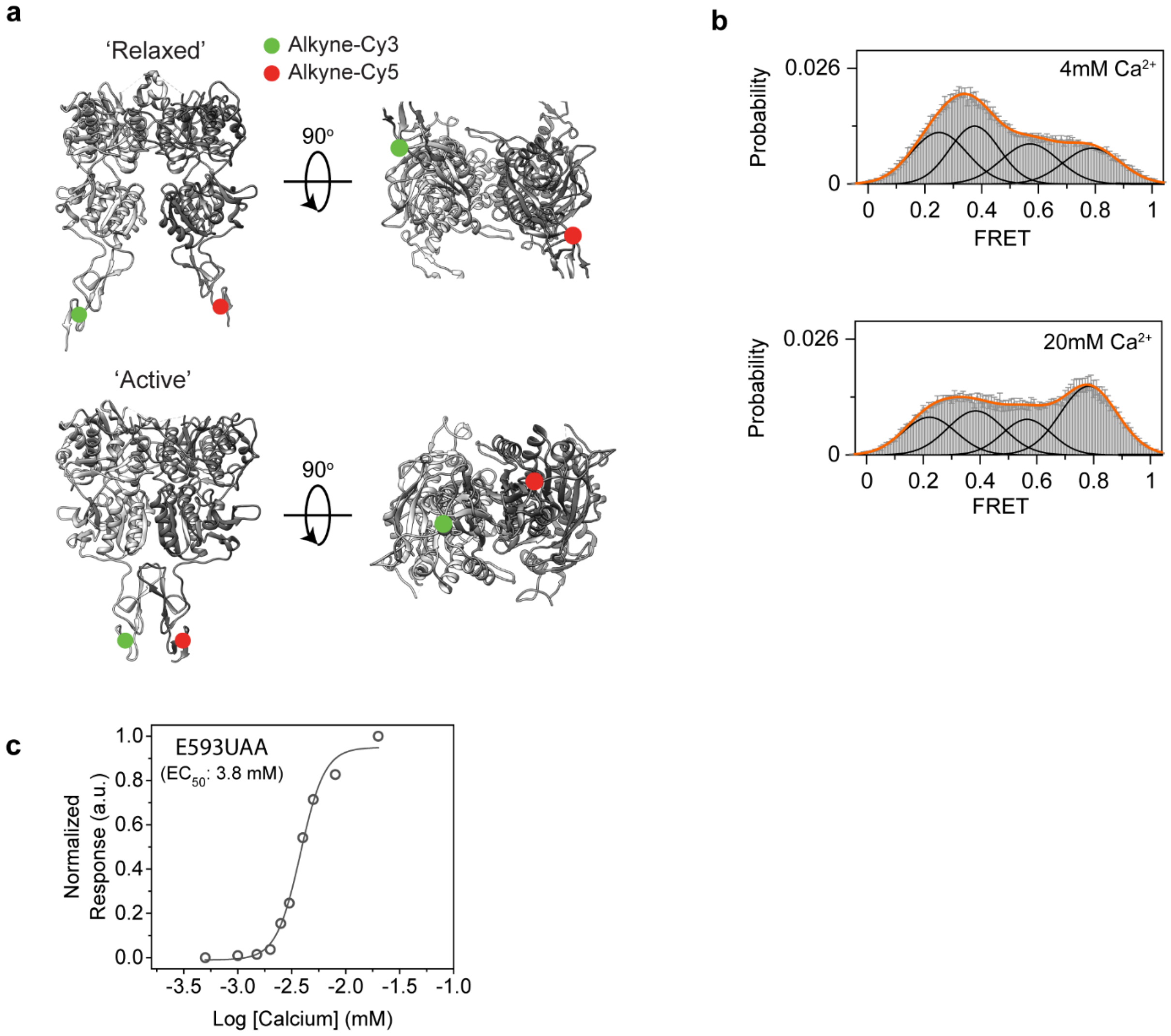

Supplementary Fig. 3 – smFRET population histograms of D451UAA and E593UAA.

a Ribbon representation of crystal structures PDB 5K5T (top) and PDB 5K5S (bottom) showing approximate location of E593UAA labelling. **b** smFRET population histogram in the presence of 4 mM Ca^{2+}, or 20 mM Ca^{2+}. Data represent mean ± s.e.m. of n=3 independent biological replicates. Histograms were fit with four single gaussian distributions (black) centered at 0.22, 0.38, 0.56, 0.78, and the cumulative fit is overlaid (orange). **c** Dose-response curve of E593UAA. Data represent n=1 independent biological replicate. Data was fit to the hill equation to calculate EC_{50}.

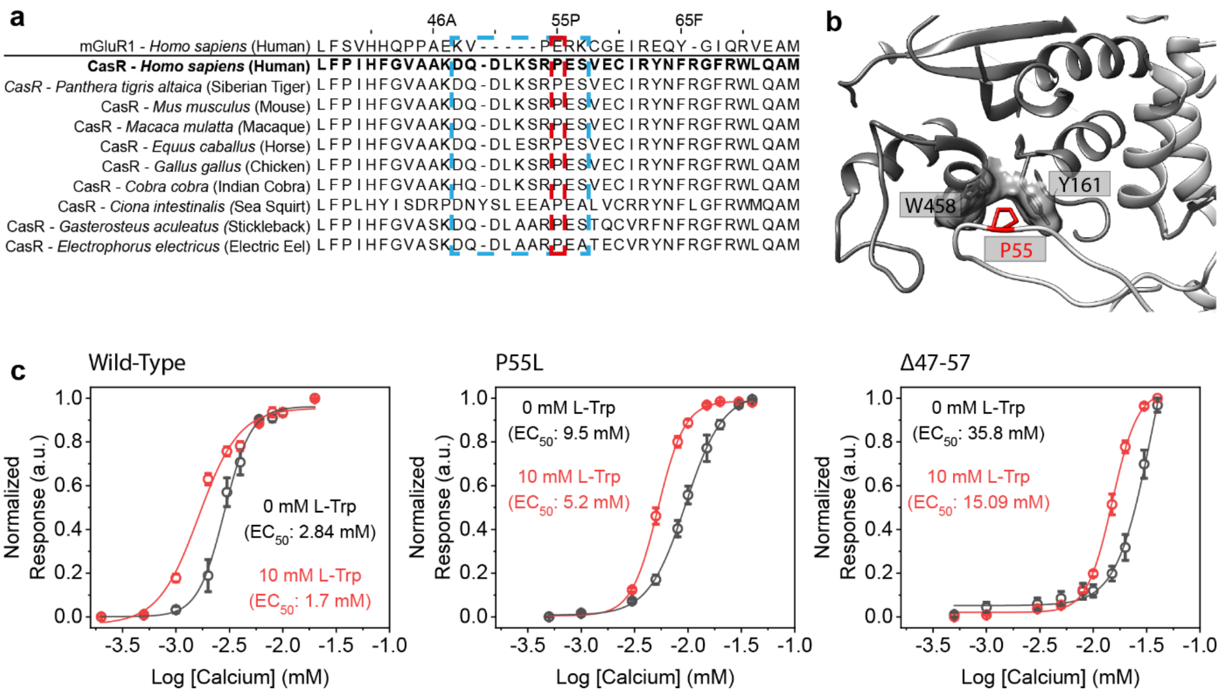

Supplementary Fig. 4 – A conserved proline makes critical contacts between protomers.

a Multiple sequence alignment of human mGluR1, human CaSR, and select CaSR orthologues. CaSR is used as reference for residue numbering. Dashed boxes indicate the interprotomer loop (blue) or the conserved proline (red). **b** Close up view of interprotomer loop of CaSR(PDB 5K5S) showing the interaction of P55 (red) and adjacent residues W458 and Y161 with surface representation. **c** Dose-response curves Wild-Type, P55L, and Δ47-57 CaSR in the absence and presence of 10 mM L-Trp. Data represent mean ± s.e.m. of n=3 independent biological replicates. Data was fit to the hill equation to calculate EC_{50}.

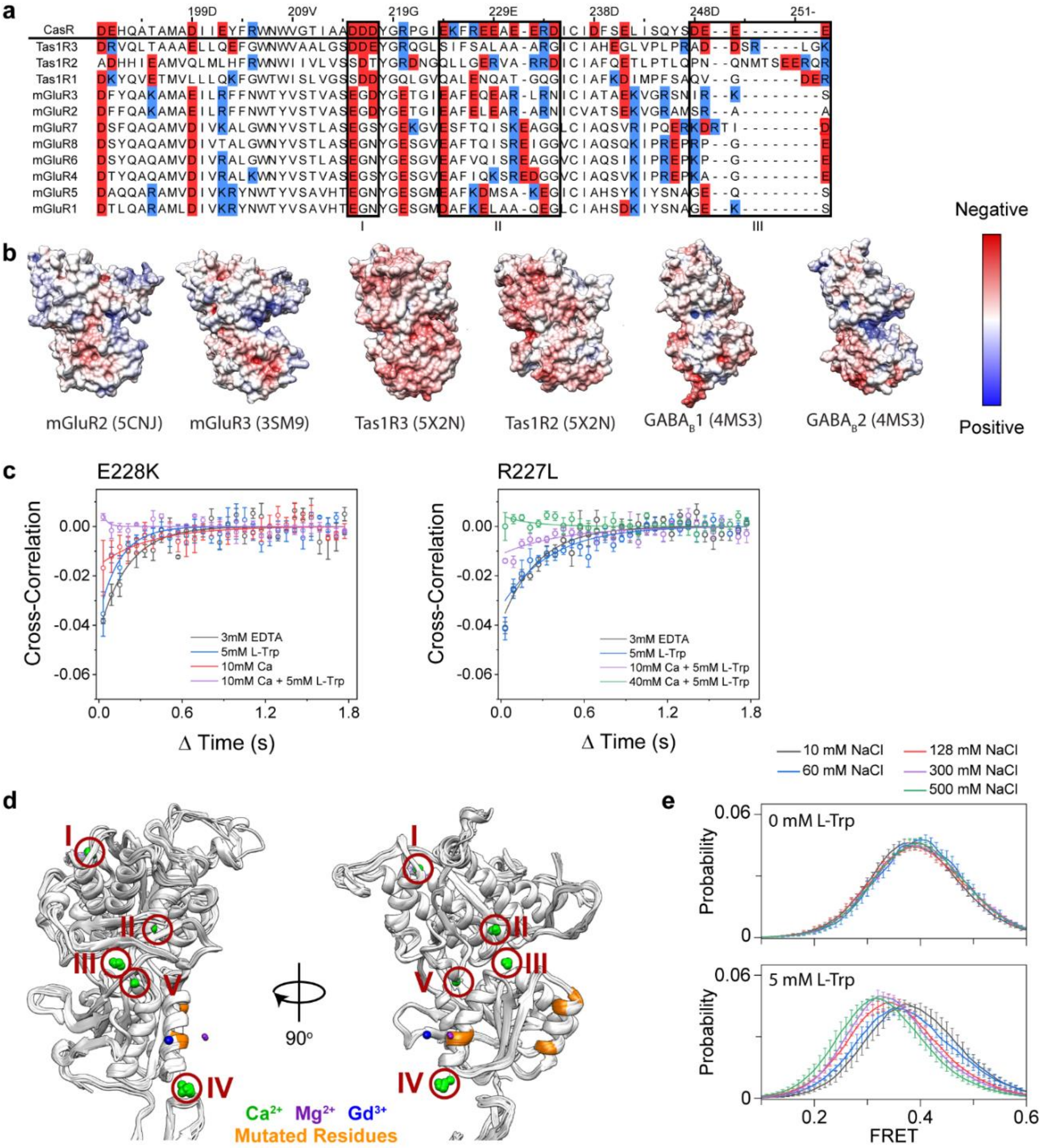

Supplementary Fig. 5 – Multiple sequence alignment and electrostatic surface potential maps of Class C GPCRS.

a Multiple sequence alignment of human CaSR and its human paralogs with negatively charged residues (red) and positively charged residues (blue) highlighted. CaSR is used as reference for residue position. Boxes indicate regions of high charge density unique to CaSR. **b** Electrostatic potential maps for mGluR2, $GABA_B1$, $GABA_B2$, mGluR3, Tas1R3, and Tas1R2. **c** Cross-correlation plot quantifying the dynamics of mutant receptors E228K (left) and R227L (right). Data represent ± s.e.m of n=3 independent biological replicates. Data was fit to a single exponential decay function. **d** Ribbon representation of CaSR structures bound to ions with Ca^{2+} binding sites I-V circled in red. Mutated residues R227, E228, E249, E251, and V258 colored orange. **e** smFRET population histograms with varying concentrations of NaCl and 0 mM L-Trp (top) or 5 mM L-Trp (bottom). Data represent ± s.e.m of n=3 independent biological replicates.

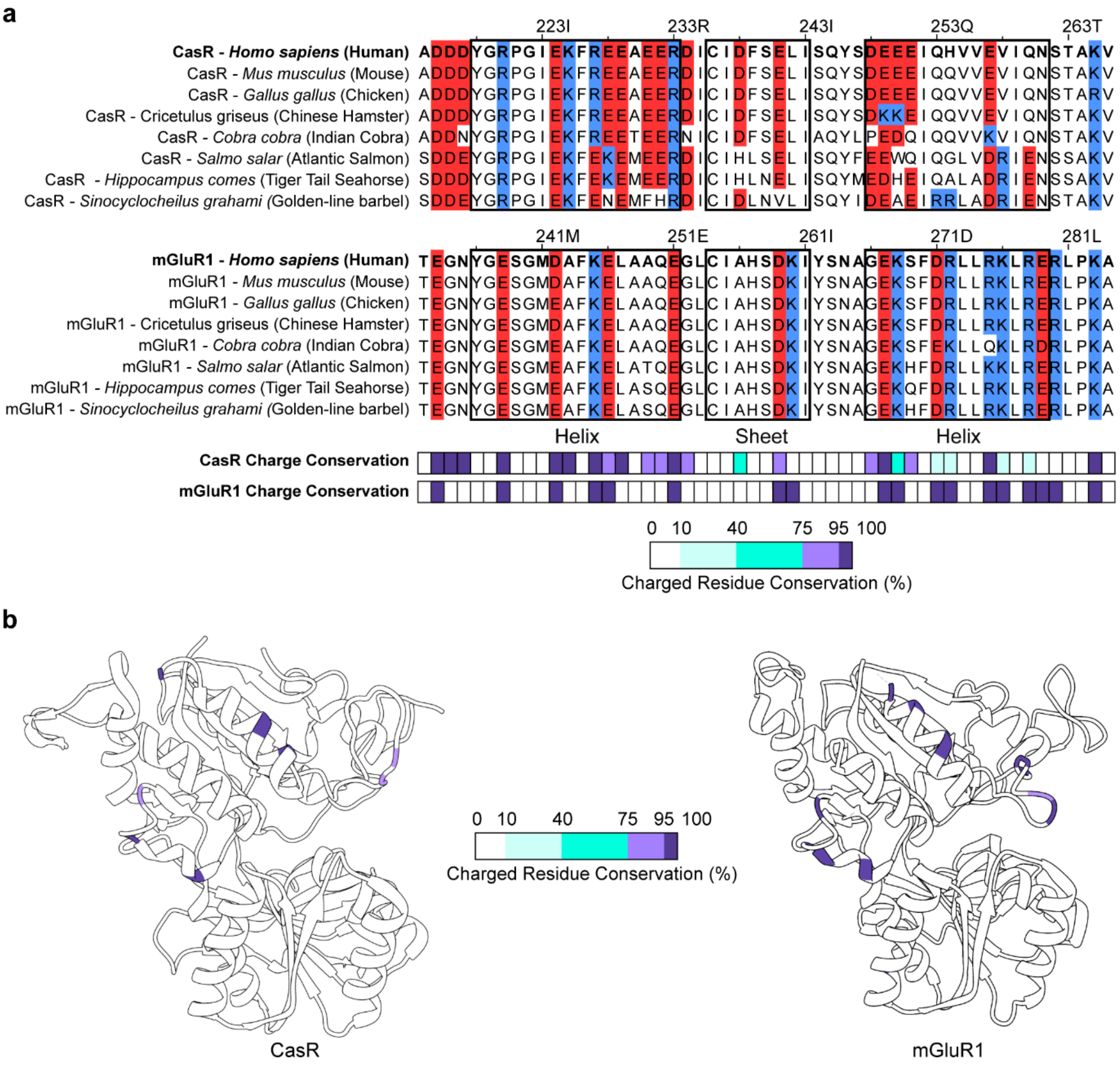

Supplementary Fig. 6 – Alignment of helix-sheet-helix motif and conservation of charged residues in upper lobe.

a Multiple sequence alignment of human CaSR with select orthologs (top), human mGluR1 with select orthologs (middle), and conservation of charged residues for each position (bottom). A conserved helix-sheet-helix structural motif in Class C lower lobe shown in boxes. Negatively charged (red) and positively charged (blue) residues are highlighted. Human sequences for CaSR and mGluR1 used as reference for residue positions. **b** Ribbon representation of CaSR (left, PDB 5K5S) and mGluR1 (right, PDB 1ISR) crystal structures displaying the conservation of charged residues for the upper lobe interface.

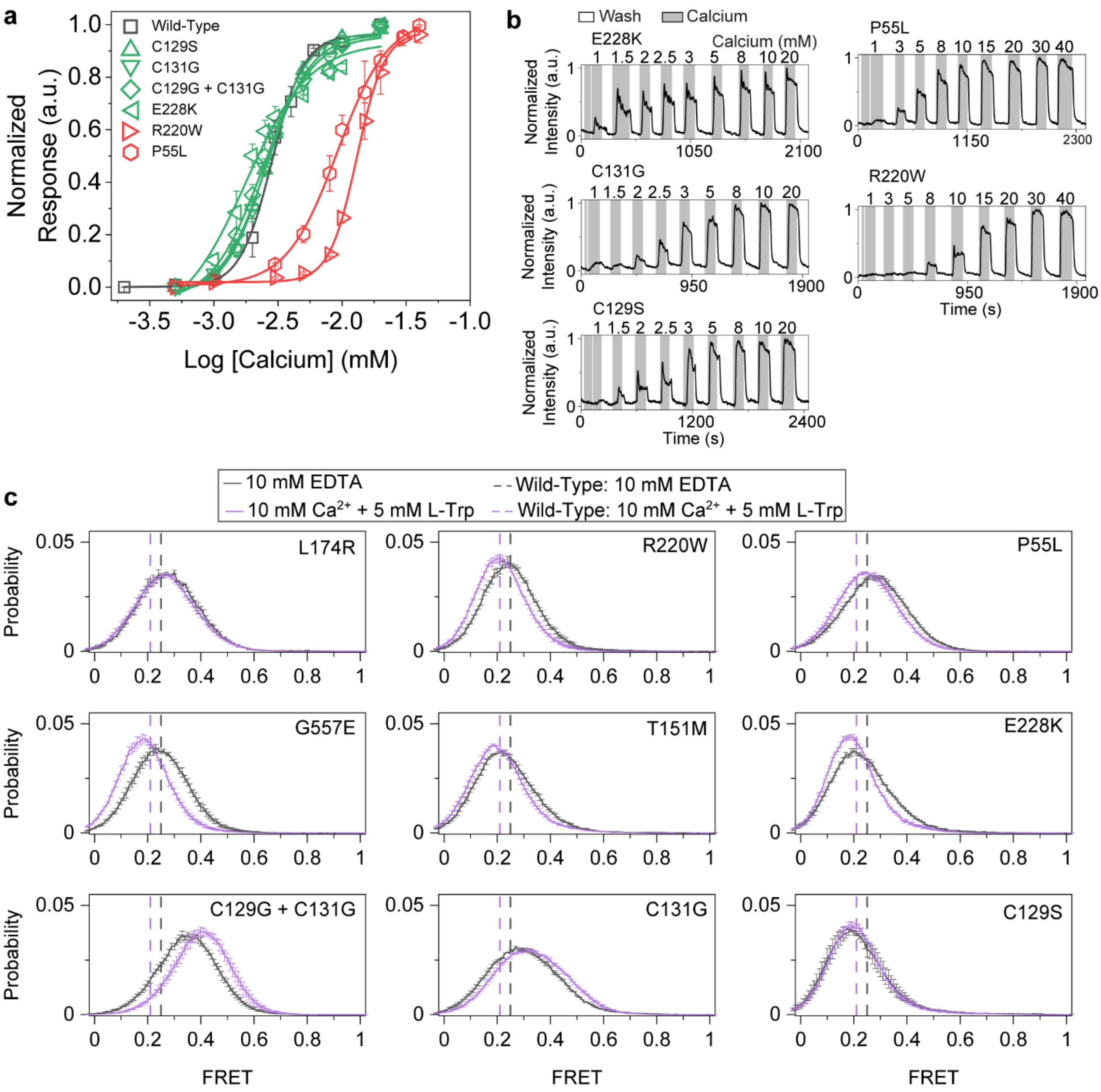

Supplementary Fig. 7 – Functional characterization smFRET histograms of CaSR mutants.

a Dose-response curve of wild-type (gray), sensitizing (green), and desensitizing (red) mutations. **b** Cell response traces showing normalized response to increasing concentrations of calcium. Shaded regions indicate times when cells were stimulated with calcium. **c** smFRET population histograms of N-Terminal SNAP CaSR mutants in the presence of 10 mM EDTA or 10 mM Ca^{2+} and 5 mM L-Trp. Dashed lines indicate centers of Wild-Type distributions for reference. Data represent mean ± s.e.m. of n=3 independent biological replicates.

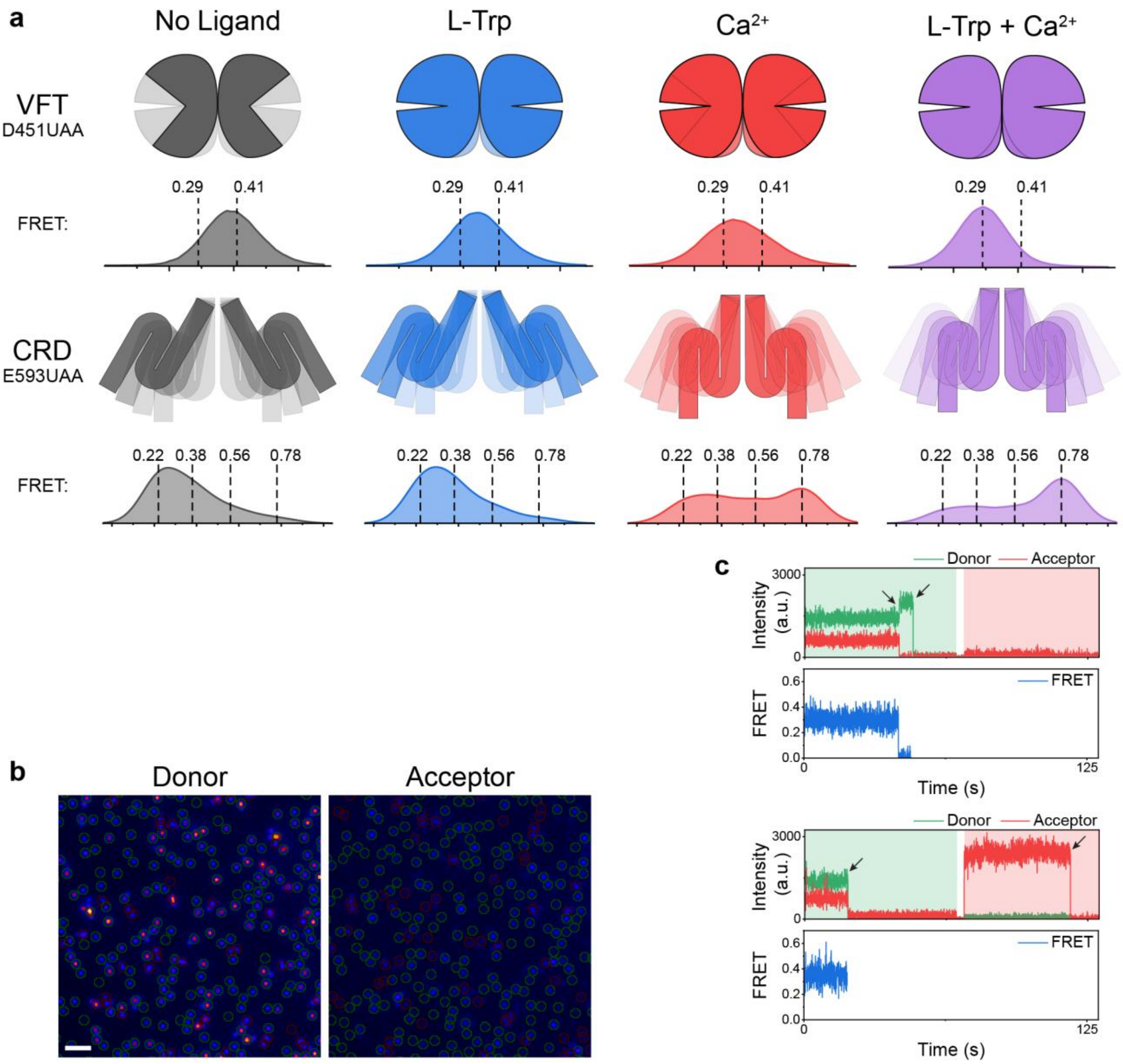

Supplementary Fig. 8 – Functional characterization smFRET histograms of CaSR mutants.

a Model schematic of CaSR showing the effect of ligands on the VFT and CRD domains and corresponding FRET distributions. **b** Sample image of smFRET movie showing the donor channel (left) and acceptor channel (right). Scale bar, 3 µM. **c** Sample single-molecule traces. Shaded regions correspond to excitation by donor (green) and acceptor (red) lasers. Arrows indicate points of single step photobleaching events that demonstrate the presence of a single donor and single acceptor fluorophore.
