# Supplementary material for: Mechanism of sensitivity modulation in the calcium-sensing receptor via electrostatic tuning
Source: Nat Commun. 2022 Apr 22;13:2194. doi: 10.1038/s41467-022-29897-y (PMC9033857; doi:10.1038/s41467-022-29897-y)
Supplement: Supplementary file 3 — Description of Additional Supplementary Files [file 41467_2022_29897_MOESM3_ESM.pdf]

Description of Additional Supplementary Files

File name: Supplementary Data 1

Description: Analysis information including particle counts for individual conditions in single-molecule FRET analysis and curve fitting parameters.
