# Supplementary material for: Mechanism of sensitivity modulation in the calcium-sensing receptor via electrostatic tuning
Source: Nat Commun. 2022 Apr 22;13:2194. doi: 10.1038/s41467-022-29897-y (PMC9033857; doi:10.1038/s41467-022-29897-y)
Supplement: Supplementary file 5 — Reporting Summary [file 41467_2022_29897_MOESM5_ESM.pdf]

nature portfolio

Corresponding author(s): Reza Vafabakhsh
Last updated by author(s): Mar 14, 2022

Reporting Summary

Nature Portfolio wishes to improve the reproducibility of the work that we publish. This form provides structure for consistency and transparency in reporting. For further information on Nature Portfolio policies, see our Editorial Policies and the Editorial Policy Checklist.

Statistics

For all statistical analyses, confirm that the following items are present in the figure legend, table legend, main text, or Methods section.

n/a	Confirmed	
☐	☒	The exact sample size (*n*) for each experimental group/condition, given as a discrete number and unit of measurement
☐	☒	A statement on whether measurements were taken from distinct samples or whether the same sample was measured repeatedly
☒	☐	The statistical test(s) used AND whether they are one- or two-sided<br>*Only common tests should be described solely by name; describe more complex techniques in the Methods section.*
☒	☐	A description of all covariates tested
☒	☐	A description of any assumptions or corrections, such as tests of normality and adjustment for multiple comparisons
☐	☒	A full description of the statistical parameters including central tendency (e.g. means) or other basic estimates (e.g. regression coefficient) AND variation (e.g. standard deviation) or associated estimates of uncertainty (e.g. confidence intervals)
☒	☐	For null hypothesis testing, the test statistic (e.g. *F*, *t*, *r*) with confidence intervals, effect sizes, degrees of freedom and *P* value noted<br>*Give P values as exact values whenever suitable.*
☒	☐	For Bayesian analysis, information on the choice of priors and Markov chain Monte Carlo settings
☒	☐	For hierarchical and complex designs, identification of the appropriate level for tests and full reporting of outcomes
☒	☐	Estimates of effect sizes (e.g. Cohen's *d*, Pearson's *r*), indicating how they were calculated

Our web collection on statistics for biologists contains articles on many of the points above.

Software and code

Policy information about availability of computer code

Data collection	Single-molecule and cell-based TIRF data acquisition was done using smCamera (version 1.0), which is previously published software and is available at (http://ha.med.jhmi.edu/resources/).<br><br>Confocal image data acquisition was done using Zen (Blue Edition; 2.3 system).
Data analysis	Softwares used for analysis include: smCamera (version 1.0; http://ha.med.jhmi.edu/resources/), OriginPro 2018b (b 9.5.5.409), MATLAB R2020b (9.9.0.1467703), Deepview (4.1.0), Chimera (1.15), Rstudio (1.3.1093), R (4.0.5), Python (3.7) and Fiji (1.53C).<br><br>ebFRET (version 1.1.1; last edited 12-17-2014) is an open source MATLAB package used to generate idealized traces for single-molecule data.<br><br>The custom codes for data analysis are available from the corresponding author upon request.

For manuscripts utilizing custom algorithms or software that are central to the research but not yet described in published literature, software must be made available to editors and reviewers. We strongly encourage code deposition in a community repository (e.g. GitHub). See the Nature Portfolio guidelines for submitting code & software for further information.

Data

Policy information about availability of data

All manuscripts must include a data availability statement. This statement should provide the following information, where applicable:

- Accession codes, unique identifiers, or web links for publicly available datasets
- A description of any restrictions on data availability
- For clinical datasets or third party data, please ensure that the statement adheres to our policy

A subset of single molecule data o has been deposited at https://doi.org/10.7910/DVN/PKR9SD. The plasmids used in this study are available from the corresponding author upon request. The PDB accession codes for structures used in this paper are: 5K5S, 5K5T, 7DTW, 7DTU, 7DTV, 7DTT, 1ISR, 1EWT, 3LMK, 6N51, 5FBK, 5FBH, 7M3F, 7M3E, 7E6T, 7DD7, 7DD6, 7DD5, and 7M3G. Ensembl gene IDs used to search for homologous proteins are ENSG00000036828 and ENSG00000164082. Source data are provided with this paper. All the materials and data reported in this study are available from the corresponding author upon reasonable request.

Field-specific reporting

Please select the one below that is the best fit for your research. If you are not sure, read the appropriate sections before making your selection.

☒ Life sciences ☐ Behavioural & social sciences ☐ Ecological, evolutionary & environmental sciences

For a reference copy of the document with all sections, see nature.com/documents/nr-reporting-summary-flat.pdf

Life sciences study design

All studies must disclose on these points even when the disclosure is negative.

Sample size	No statistical method was used to predetermine sample sizes. A sample size of more than 300 molecules per condition was obtained and found to adequately sample molecule behavior. This sample size was chosen. Increasing the sample size did not change our results, suggesting our sample size was sufficiently large. A standard replicate size of n=3+ was used. All the biological samples and reagents were made fresh for each experiment.
Data exclusions	Details of exclusion criteria were described in detail in the manuscript, were pre-established, and were applied uniformly to all samples. For cell-based experiments, all cell ROIs that showed a substantial drift in signal or did not respond to stimulus were excluded from analysis. During idealized trace fitting of single-molecule data, traces for which the HMM fit did not converge (for example long blinking events or non anti-correlated intensity fluctuations) or fit a single state (for example an intermediate state may be fit to very dynamic traces) were omitted from downstream analysis.
Replication	All experiments were repeated n=3 to 6 times and in all cases the attempts were successful and consistent results were obtained. No biological sample was used in more than one set of experiments.
Randomization	No sample randomization was performed and the experimental condition were systematic. The samples were not divided into separate experimental groups.
Blinding	Blinding of all the data was not possible as sample preparation and experiments were performed by a single researcher, and fresh preparation of reagents prior to collection was necessary. A subset of data was blindly analyzed to ensure no bias in analysis for different conditions for each individual and the results were identical.

Reporting for specific materials, systems and methods

We require information from authors about some types of materials, experimental systems and methods used in many studies. Here, indicate whether each material, system or method listed is relevant to your study. If you are not sure if a list item applies to your research, read the appropriate section before selecting a response.

Materials & experimental systems

n/a	Involved in the study	
☐	☒	Antibodies
☐	☒	Eukaryotic cell lines
☒	☐	Palaeontology and archaeology
☒	☐	Animals and other organisms
☒	☐	Human research participants
☒	☐	Clinical data
☒	☐	Dual use research of concern

Methods

n/a	Involved in the study	
☒	☐	ChIP-seq
☒	☐	Flow cytometry
☒	☐	MRI-based neuroimaging

Antibodies

Antibodies used	THE™ DYKDDDDK Tag Antibody [Biotin], Mouse. GenScript Cat. No. A01429 (Immunogen: A synthetic peptide (DYKDDDDK) coupled to KLH)<br>Biotin Anti-GFP antibody, Goat. From Abcam Cat. No. ab6658 (Recombinant full length protein corresponding to GFP aa 1-246)
Validation	All antibodies were validated by the manufacturers indicated above. The validation of these antibodies can found in the respective vendor websites.<br>Quality control tests for reactivity were performed by the manufacturer and showed an ELISA titer ≥1:20,000 and also passed western blot validation by showing specific reactivity for GFP or DYKDDDDK-tagged proteins. For our purposes the anti-FLAG and anti-GFP antibodies were used for pull-down of receptors in the single-molecule FRET assay. Antibodies did not pull-down fluorescently labeled receptors without the FLAG tag or mEGFP.

Eukaryotic cell lines

Policy information about cell lines

Cell line source(s)	HEK-293T cells were purchased from Millipore-Sigma for this study.
Authentication	Cell lines were not authenticated.
Mycoplasma contamination	Cell lines were not tested for mycoplasma contamination.
Commonly misidentified lines<br>(See ICLAC register)	No commonly misidentified cell lines were used in the study.
